# Influence of Wooden Sawdust Treatments on Cu(II) and Zn(II) Removal from Water

**DOI:** 10.3390/ma13163575

**Published:** 2020-08-13

**Authors:** Zdenka Kovacova, Stefan Demcak, Magdalena Balintova, Cocencepcion Pla, Inga Zinicovscaia

**Affiliations:** 1 Faculty of Civil Engineering, Institute of Environmental Engineering, Technical University of Kosice, Vysokoskolska 4, 042 00 Kosice, Slovakia; zdenka.kovacova.2@tuke.sk (Z.K.); magdalena.balintova@tuke.sk (M.B.); 2 Department of Civil Engineering, University of Alicante, Carretera de s/n, 03690 Alicante, Spain; c.pla@ua.es; 3 Joint Institute for Nuclear Research, Joliot-Curie Str. 6, 1419890 Dubna, Russia; zinikovskaia@mail.ru; 4 Horia Hulubei National Institute for R&D in Physics and Nuclear Engineering, 30 Reactorului Str., MG-6, Bucharest, 077125 Magurele, Romania

**Keywords:** heavy metals, sorption, wooden sawdust, alkaline modification, Freundlich isotherm, Langmuir isotherm

## Abstract

Organic waste materials and semi-products containing cellulose are used as low-cost adsorbents that are able to compete with conventional sorbents. In addition, their capacity to bind heavy metal ions can be intensified by chemical treatments using mineral and organic acids, bases, oxidizing agents, and organic compounds. In this paper, we studied the biosorption capacity of natural and modified wooden sawdust of poplar, cherry, spruce, and hornbeam in order to remove heavy metals from acidic model solutions. The Fourier transform infrared spectroscopy (FTIR) spectra showed changes of the functional groups due to the alkaline modification of sawdust, which manifested in the considerably increased intensity of the hydroxyl peaks. The adsorption isotherm models clearly indicated that the adsorptive behavior of metal ions in treated sawdust satisfied not only the Langmuir model, but also the Freundlich model. The adsorption data obtained for studied sorbents were better fitted by the Langmuir isotherm model for both metals, except for spruce sawdust. Surface complexation and ion exchange are the major mechanisms involved in metal ion removal. We investigated the efficiency of the alkaline modified sawdust for metal removal under various initial concentrations of Cu(II) and Zn(II) from model solutions. The highest adsorption efficiency values (copper 94.3% at pH 6.8 and zinc 98.2% at pH 7.3) were obtained for poplar modified by KOH. For all types of sawdust, we found that the sorption efficiency of modified sorbents was higher in comparison to untreated sawdust. The value of the pH initially increased more in the case of modified sawdust (8.2 for zinc removal with spruce NaOH) and then slowly decreased (7.0 for Zn(II) with spruce NaOH).

## 1. Introduction

Environmental protection is an important aspect of sustainable development and has great importance for the future of living organisms on the entire planet. At present, the environment faces the threat of organic and inorganic pollutants of different origins. Industrial progress contributes to the economic development of individual countries. However, such progress is always accompanied by endangering and contamination of the environment by diverse pollutants [1].

The Slovak Republic has a rich mining history of minerals, including copper, gold, nickel, and others. The attenuation of the processing minerals at the beginning and in the middle of the 20th century supported the emergence of the extensive closing of deposits using wet conservation, i.e., flooding. Acid mine damage (AMD) is a serious environmental problem associated with mining activity caused by the oxidation of sulfide containing minerals, such as pyrite and its polymorph marcasite. AMD threatens the environment directly through polluting streams, rivers, groundwater, and natural ecosystems. The outflow from old mines has a negative impact on the pH values and increases the levels of sulphate and heavy metals [2,3,4,5].

In contrast to organic contaminants, heavy metals are non-degradable and cannot be easily biologically detoxified. They can accumulate in living organisms through the consumption of contaminated food and water [1,6,7].

Copper is one of the most expanded pollutants in the nature. Copper is a heavy metal that is toxic to living beings in large quantities; however, copper is also an essential element with an important role in the metabolism of all living beings [8]. The wide application of copper in industrial activities is due to its properties, including high electrical and thermal conductivity, good corrosion resistance, ready availability, and high recyclability. High doses of copper can cause nausea, vomiting, headache, diarrhea, respiratory difficulties, liver and kidney failure, and in the worst cases even death [9].

Zinc is the second most abundant metal in living beings after iron. Zinc is chemically active and easily forms alloys with other metals in the environment. Zinc is applied in the galvanization of steel, the preparation of alloys, and in the production of negative plates in electrical batteries. It can be used as a pigment in cosmetics, plastics, photocopier paper, wallpaper, printing inks, etc. In the rubber industry, it acts as a catalyst during processing and as a heat disperser in the final phase of manufacturing [10,11]. High concentrations of zinc in human food can cause serious health problems, including skin irritations, stomach cramps, vomiting, anemia, and nausea [12].

These contaminants mostly present in the acid mine drainage of closed copper and zinc mines [13,14,15]. Previous research [3,4,5,16] was dedicated to AMD monitoring and treatment. The highest value of contaminants was obtained in Pech shaft at a pH value 4.0 ± 0.1 [3].

Due to the negative effects of heavy metals, eliminating them from waste waters is a priority [1]. Many processes have been developed for the removal of heavy metals from contaminated waters, for instance chemical precipitation, flotation, ion exchange, coagulation and flocculation, membrane filtration, electrodialysis, and adsorption for the purpose of water treatment [17]. The advantages and disadvantages of these methods are summarized in Table 1.

The majority of these methods [7,18,19,20,21,22,23,24,25,26,27,28,29,30] are expensive, ineffective in certain concentrations, or time-consuming, and adsorption is limited by the cost of the adsorption material [1,6,7,8,9,31,32]. For these reasons, the choice of sorbent is crucial for the development of a simple, efficient, inexpensive, and environmentally friendly water purification method [33,34].

Adsorption using natural materials is another possibility to eliminate the high cost of industry-made sorbents [6]. In recent years, researchers intensively investigated the adsorption of pollutants from waters using a variety of natural (biological) materials, including agricultural by-products and waste materials from the processing of food, which can be referred to as biosorption [35,36]. Low-cost sorbents can be used for metal adsorption in either their natural form or after suitable modifications to improve their adsorption capacity [33,34,37]. Adsorbents suitable for this purpose are biodegradable and cheap compared to activated carbon or ion exchange resins [1]. These materials include corn stalk [38], tree bark [39], grain residues [40], almond shells [41], sunflower stalks [42], peat [43], peanut husk [44], tea leaves [45], sawdust [46], and others with the capacity for water treatment.

Wooden sawdust [47], a by-product of the wood industry, was first investigated as a sorbent to remove Cu(II) from wastewater 20 years ago. As the research continued [1,6,7,9,31,32,33,34,35], sawdust was found to be one of the most widespread, inexpensive materials capable of removing pollutants from wastewater. Shukla et al. [48] confirmed sawdust as an efficient sorbent for the removal of dyes, oil, salts, heavy metals, etc.

The cell walls of wooden sawdust consist of crude fibers, acid detergent fibers (containing various organic compounds, such as lignin, cellulose, and hemicelluloses), and many hydroxyl groups, such as phenolic compounds and tannins, which are useful for binding heavy metals ions through the mechanism of ion exchange and adsorption. The ion exchange processes are accompanied by intensive changes of the pH values of the water solution [46,47,48,49,50,51]. The mechanism of the sorption process includes the binding of the heavy metal ions with the hydroxyl, metyl, carboxyl, and amide functional groups in wooden sawdust. Other elements that are also contained in sawdust, such as potassium, sodium, and calcium, can participate in the ion exchange process. Complexation and microprecipitation processes can also be involved in sorption. Heavy metals in reactions with sawdust accumulate in the secondary septa of the wood, which is rich in cellulose and poor in lignin. The lignin content of hardwood (cherry and hornbeam) is usually 18–28% and 25–35% in softwood (poplar, spruce) [46,50,52].

The surface properties of sawdust [48] show that the binding capacity of heavy metals is connected with the presence of alcohol and phenolic functional groups. Various low-cost sorbents, such as rice husks, mandarin peels, rubber leaves, walnut shells, and pine bark, showed improvements in the adsorption capacity after alkali modification [53,54]. A higher sorption capacity for Pb(II) was obtained following the modification of Dibetou sawdust (*Lovoa trichilioides*) with nitric acid and sodium hydroxide due to the ensured activation of the adsorption sites, by minimizing the content of the lignin and hemicelluloses contained therein, and by increasing the porosity of the adsorbent matrix and its specific surface [1]. Additionally, KOH modification increased the aromatic and oxygen-containing functional groups by two to three times, resulting in an increase of the sorption capacity compared to unmodified sorbents, such as coir and jute fibers, teakwood sawdust, and groundnut shells of Pb(II), as assessed by Shukla and Pai [49]. Alkaline modification had a positive effect on the improvement of sorption capacity [1,33,34,38,53,54]; however, there are only few references regarding the modification of sawdust.

The objective of the present study was to investigate the possible use of sawdusts an alternative adsorbent materials for the removal of Zn(II) and Cu(II) from waste solutions by testing them with synthetic sawdusts. Wooden sawdusts, including cherry (*Prunus avium*), poplar (*Populus nigra*), hornbeam (*Carpinus betulus*), and spruce (*Picea abies*), obtained from local sources were used as sorbents in the experiments. The sorptive properties of natural sawdusts were compared with those of sawdust modified by 1 M sodium hydroxide or potassium hydroxide. The starting pH in all solutions was adjusted to 4.0 due to approaching the AMD waters. We used Fourier transform infrared spectroscopy (FTIR) spectrometry to determine the functional groups involved in the sorption process responsible for metal binding. The Langmuir and Freundlich models were used to describe the experimental data and to explain the probable mechanism of sorption.

## 2. Materials and Methods

### 2.1. Sorbent Preparation

In the experiments, we used natural and modified sawdusts. Cherry, poplar, hornbeam, and spruce sawdust obtained from locally available wood were sieved, and only fractions with a particle size lower than 2.00 mm were used in the experiments. Based on published studies [34,55,56], both types of modification were realized with a 1 M solution of sodium hydroxide or 1 M solution of potassium hydroxide for the removal of residual lignin and the creation of new sorption sites on the surface of the materials. The modification procedure consisted of 20 g of wooden sawdust mixed with 200 mL of NaOH or KOH solution for 24 h without stirring. After this time, the sawdust was filtered through filter paper, washed several times with distilled water, and dried in an oven at 45 ± 5 °C. 

### 2.2. Synthetic Solutions

Copper or zinc stock solutions (10,000 mg/L) were prepared by dissolving a given amount of sulphate salts (Merck, Darmstadt, Germany) in distilled water. Lower concentrations (10, 30, 50, 70, 90, 110, 130, and 150 mg/L) were prepared by diluting the stock solutions with distilled water. Sulfuric acid was used to adjust the starting pH values in each copper or zinc solution independently to a value of 4.0 to better observe the final pH values and the removal efficiency between the natural and modified sorbent materials.

### 2.3. Sorption Experiments

The sorption process depends on several parameters, including the initial pH, initial concentration, adsorbent dosage, temperature, ionic strength, and particle size [54,57]. In this experiment, we measured the adsorption capacity and pH as important parameters in the adsorption process.

The sorption experiments were conducted by adding 0.5 g of wooden sawdust to flasks containing 50 mL of synthetic copper or zinc solution at concentrations of 10, 30, 50, 70, 90, 110, 130, and 150 mg/L. The flasks were left in the laboratory for 24 h at a constant temperature (22 ± 2 °C) without any stirring. This contact time was sufficient to achieve sorption equilibrium of the zinc and copper ions in model solutions. After this, the solution was filtered, then the pH and the concentration of Cu(II) or Zn(II) in the filtrate were measured. The concentrations of metal ions were measured using the colorimetric method with an appropriate reagent [58].

We used a DR 890 colorimeter (Hach Lange, Loveland, CT, USA) for the determination of Cu(II) and Zn(II) in aqueous solutions. The copper concentration was determined using the Bicinchoninate method using a powder pillow adapted from Nakano [59]. Copper in the sample reacted with a CuVer 1 copper reagent (Hach Lange, Loveland, CT, USA), which contained salt of bicinchoninic acid. After 2 min of reaction time, a purple-colored complex in proportion to the copper concentration was formed [60].

The concentration of zinc was measured using the Zircon method adapted from standard methods for the examination of water and wastewater approved by the United States Environmental Protection Agency (USEPA) (Standard Methods for the Examination of Water and Wastewater) for analysis with the ZincoVer 5 reagent (Hach Lange, Loveland, CT, USA) [61]. Zinc in the sample reacted with cyanide in the ZincoVer 5 reagent after adding cyclohexanone, then the zinc was released and reacted with the 2-carboxy-2′-hydroxy-5′-sulfoforamazyl benzene (zincon) indicator. After 3 min of reaction, a blue color proportional to the zinc concentration was formed [60].

The pH measurements of the filtrate were performed with a FiveGo pH meter FG2 (Mettler, Weilheim, Germany). The pH meter was standardized using buffer solutions with pH values of 4.01 and 7.00. All filtrations were realized with quantitative F11P folded filter paper.

In addition, the FTIR spectra of the adsorbent materials were performed using a Bruker Alpha Platinum-ATR spectrometer (BRUKER OPTICS, Ettingen, Germany). There were performed for a total of 24 scans of the modified and natural sawdusts. The spectra were recorded in the range of 4000 to 400 cm^−1^.

The efficiency of the sorption process, *E,* expressed as a percentage of the adsorbed metal compared to the initial metal concentration; and the amount of metal ions sorbed per specific amount of adsorbent, *q* (mg/g), were calculated by following Equations (1) and (2), respectively:

(1) $$E=(\left(C_{0}-C\right)/C_{0})\times 100$$

(2) $$q=\left(C_{0}-C\right)\times V/m$$

where *C_0_* is the initial metal concentrations in mg/L, *C* is the final metal concentration after ion sorption in mg/L, *m* is the mass of sawdust in g, and *V* is the volume of the aqueous solution in L [62,63,64].

### 2.4. Adsorption Models

In the present study, the equilibrium data for copper and zinc removal using wooden sawdust was described using two models, the Langmuir and Freundlich models, with the aim of determining a better fitting isotherm [65].

#### 2.4.1. Langmuir Model

The Langmuir model [66] assumes the adsorption of molecules forming a monolayer. It is described as homogeneous, assuming that all adsorption sites have the same adsorption affinity and adsorption at one site does not affect adsorption at the adjacent site. The isotherm equation is given as Equation (3):

(3) $$q=q_{m}\times K_{L}\times C/1+K_{L}\times C$$

where *q* is the amount of metal ions adsorbed per specific amount of adsorbent in mg/g, *C* is the equilibrium concentration in mg/L, *q_m_* is the quantity of heavy metal ions necessary to form a single monolayer on a unit mass of sorbent in mg/g, and *K_L_* is the Langmuir equilibrium constant, which is related to the seeming energy of adsorption.

The separation factor *R_L_* can be described as Equation (4):

(4) $$R_{L}=\frac{1}{1+K_{L}\times C_{0}}$$

This indicates the isotherm shape and whether the adsorption is irreversible (*_RL_* = 0), favorable (0 < *R_L_* < 1), linear (*R_L_* = 1), or unfavorable (*R_L_* > 1).

#### 2.4.2. Freundlich Model

The Freundlich model [67] was considered before to be an empirical one. This model is applicable to the sorption process that takes place on heterogeneous surfaces [65]. It assumes that at different concentrations the ratio between the amounts of solute adsorbed to a given weight of adsorbent per concentration of the solute in the solution is not constant. By increasing the scale of the process, the heat of adsorption is reduced in many systems. The equation of this model is given as Equation (5):

(5) q=KF×C1n

where *K_F_* and *n* are Freundlich equilibrium constants. *K_F_* indicates the relative adsorption capacity of the adsorbent related to the bonding energy and *n* is the heterogeneity factor or Freundlich coefficient, which represents deviation from linearity of adsorption. The Freundlich equation is used for the description of the sorption process in aqueous environments [64].

## 3. Results and Discussion

In this study, natural and modified wooden sawdusts as sorbents were used for zinc and copper removal from synthetic solution. Fourier transform infrared spectroscopy (FTIR) is a useful technique for analyzing the chemical and structural changes that occur in wooden materials and for determining active sites that exist in the surface structures of adsorbents [68].

### 3.1. FTIR Spectra

**Figure 1:**
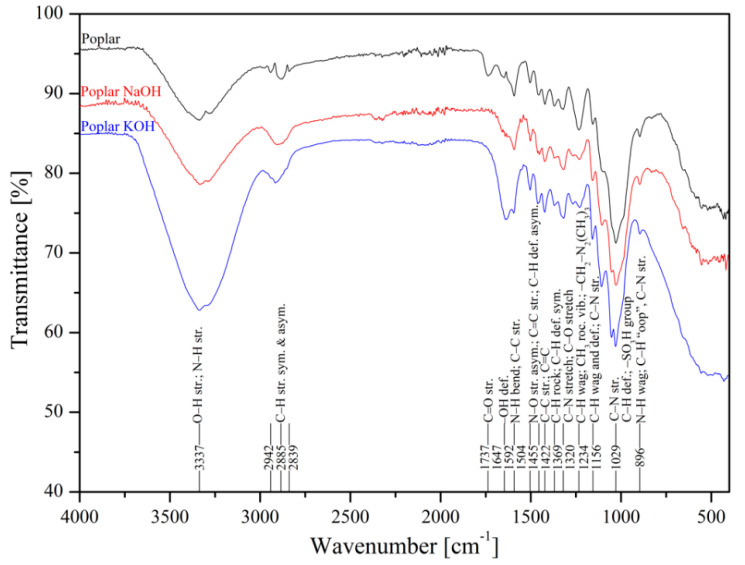
Infrared spectra of natural and treated poplar wooden sawdusts.

**Figure 2:**
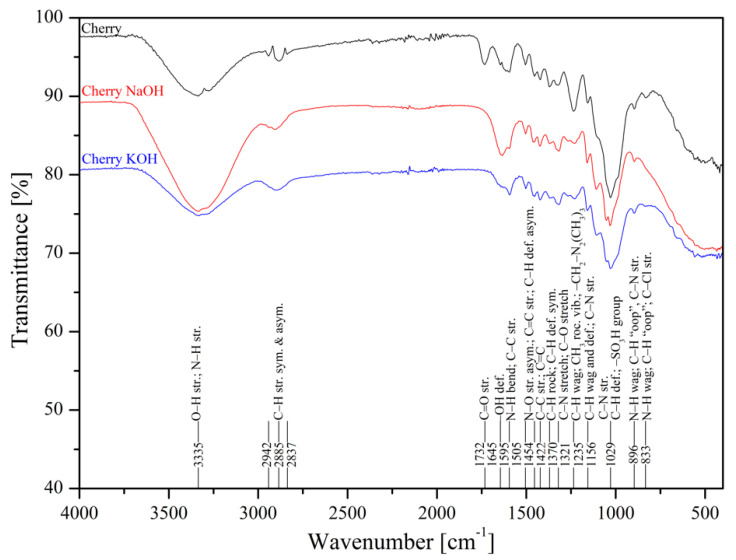
Infrared spectra of natural and treated cherry sawdusts.

The metal uptake from contaminated aquatic environments by organic adsorbents as sawdust is closely linked to the surface structure where functional groups are present, such as –OH, –COOH, –NH, –NH_2_, and –NH_3_ [50]. FTIR spectroscopy of poplar, spruce, cherry, and hornbeam sawdusts was used for determination of the functional groups. The IR spectra of sawdust (natural and alkaline treated wooden) are shown in Figure 1 (poplar) and Figure 2 (cherry), which summarize the detailed band assignments of the studied sawdust functional groups according to the literature [35,69,70,71].

The alkaline treatment significantly intensified the wide peak of the strong broad −OH stretching at wavenumber 3337 cm^−1^. The treatment with NaOH led to intensification of hydroxyl functional groups for cherry (Figure 2), spruce (Figure S1), and hornbeam (Figure S2) sawdusts, the spectra for which were almost identical. The treatment with KOH considerably intensified the –OH functional groups in poplar sawdust (Figure 1). In poplar wood, the sawdust presented different deacetylation processes, which reduced the water solubility of O-acetyl-galactoglucomannans and O-acetylglucuronoxylans from chemicelluloses, and increased the adsorption of these polymers onto cellulose fibers. This phenomenon resulted in changing the structure of the poplar sawdust, which possibly caused an increase in the number or the order of the sorption sites on the sawdust surface and increased the adsorption capacity [55]. Differences in comparisons of spectra at wavenumbers of 3000 to 2800 cm^−1^ were also revealed. The asymmetric C–H stretching vibration was rearranged to symmetric, where triple peaks were aligned and centered at wavenumber 2885 cm^−1^.

### 3.2. Removal Efficiency

The initial concentrations of heavy metals have a significant effect on the adsorption capacity of sawdust, as a certain mass of the sawdust can sorb only a certain amount of the contaminant. The higher the concentration of the copper in the solution, the less able the sawdust is to eliminate it from the solution. At higher concentrations of pollutants, the fractional adsorption is low [72]. To determine the adsorption capacity of natural and modified sawdust, sorption experiments were performed with different initial concentrations (10, 30, 50, 70, 90, 110, 130, and 150 mg/L) of copper ions at the initial value of pH = 4.0.

Sciban et al. [73] noted that sawdust leaches some organic materials into water during the process of biosorption. Demcak [50] confirmed that alkali-modified sawdust reduced the release of organic matter into the synthetic solutions.

**Figure 3:**
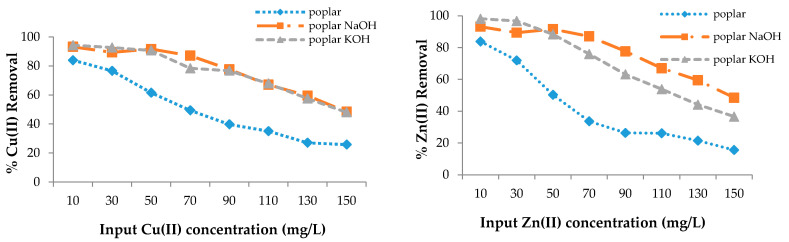

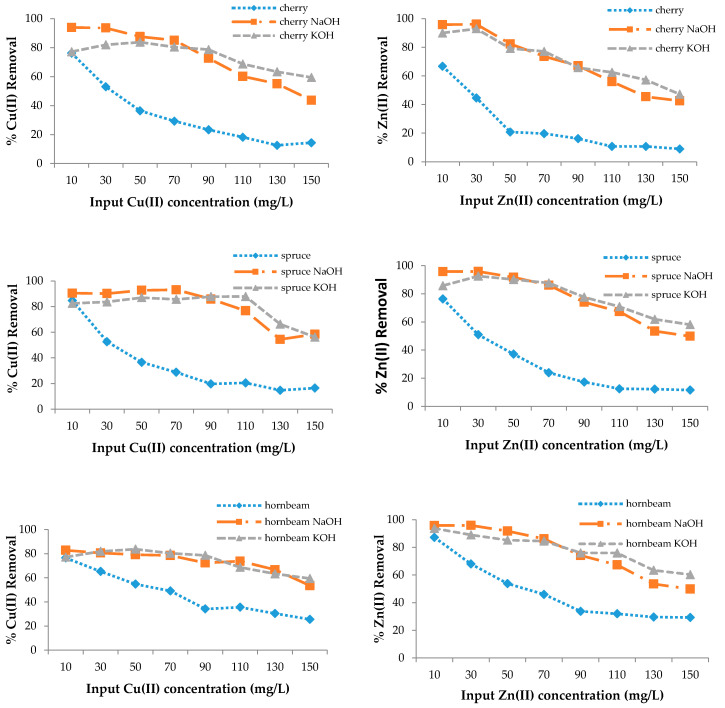
The removal efficiency for Cu(II) and Zn(II) with wooden sawdust.

The adsorption efficiency of poplar, cherry, spruce, and hornbeam sawdust for copper removal is presented in Figure 3. The sorbents had different sorption abilities for the metal ions; however, modification enhanced the sorption efficiency for both heavy metal ions. As the sorption process takes place on the surface of the wooden sawdust, its alkali modification can strongly affect the adsorption capacity of this material [63]. The maximum efficiency for all sawdusts was achieved at the lowest copper concentration of 10 mg/L, while the lowest efficiency was achieved at the highest concentration, because the ion removal efficiency is lower with increasing concentrations, despite the fact that the sawdust adsorbs the same amount of metal ions. 

In this case, the ability to adsorb the metal ions is the same. FTIR spectra confirmed that alkali modification increased the -OH groups, which resulted in an increase of the sorption properties. At 150 mg/g Cu(II) concentration in solution, the removal efficiency was approximately 1.8 and 3.5 times higher for the modification of both poplar and hornbeam, respectively; about 4 times higher for cherry KOH; 3 times higher for cherry NaOH; 2 times higher for hornbeam NaOH; and 2.3 times higher for hornbeam KOH. For zinc removal at 150 mg/L, the efficiency for spruce was 4.9 times higher for KOH, 4.2 times higher for spruce NaOH, 2 times higher for hornbeam KOH, 1.7 times higher for NaOH, 2.3 times higher for poplar KOH, 3 times higher for NaOH, 5.2 times higher for cherry NaOH, and 4.7 times higher for cherry KOH. 

The improvement in adsorption capacity after alkaline modification of sawdust was confirmed by other authors.

Memon et al. [51] performed the experiment with Cedrus deodar sawdust for the removal of Cd (II). The modification of the sawdust with 1 M NaOH for 120 min significantly affected the adsorption capacity, with the treated sawdust exhibiting greater adsorption capacity (more than 97% in 8 min) compared to the untreated one. Moreover, the alkali modification enhanced the stabilization of the wooden adsorbent, making its separation from the solution easier.

Sciban et al. [55] determined that the alkaline modification (1% of NaOH for 2 h at 20 °C) of softwood (poplar and fir) improved the adsorption capacities for Zn(II) and Cu(II), particularly for zinc. The adsorption efficiency of the modified sorbent was 2.5 to 5 times higher than the natural efficiency for copper ions and approximately 15 times higher for zinc ions.

Ofomaja et al. [74] treated pine cones with NaOH at different concentrations (0.01, 0.05, 0.10, 0.15 M). They determined that Cu(II) removal increased with NaOH modification and higher NaOH concentration. At the sorbent mass of 0.4 g of pine cone sawdust, the removal efficiency for natural sorbent was 62.56%, while for the 0.15 M modified sorbent this was 99.84%. The optimum pH value was 5 for modified and natural pine cones.

In the case of copper and zinc removal from real wastewater, Sciban [64] determined that that the absorption values from the model solution on poplar sawdust for copper and zinc were approximately equal. In the real wastewater only cadmium was significantly lower from the wastewater, as in the synthetic solution. Keränen el al. [75] removed nickel with pine sawdust from mine wastewater and found that the sorption behavior with synthetic solutions did not necessarily correlate well with real wastewaters, determining that further research is needed. 

### 3.3. Effect of pH

The initial pH was confirmed as an important parameter in the sorption process in heavy metal removal due to determining the surface charge of sawdust and the degree of ionization of the solution [48,56,57,63,76].

**Figure 4:**
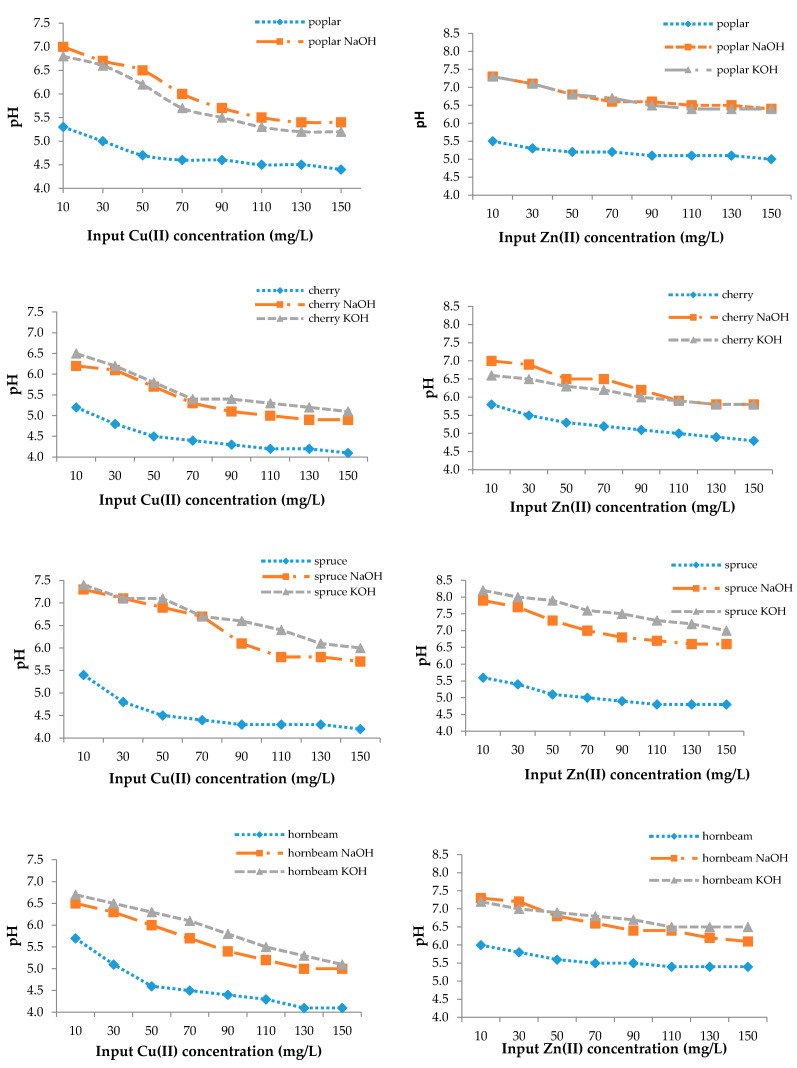
The final pH values.

From Figure 4, the change of pH from the initial value was 4.0. The pH values of natural sawdusts increased but not greatly, as in the case of the modified ones, which is the result of an increasing number of adsorption sites or their accessibility or precipitation in microspores, which can improve the adsorption efficiency as well (Figure 3) [77].

The adsorption mechanism can be explained as the heavy metal uptake increasing with increasing pH in a certain range and up to a certain value, then decreasing with additional pH increases. According to the mechanism, sorption simultaneously decreases the pH and the H^+^ release and competes with metal cations for sorption sites during the process [54,76]. At low pH, the concentration of positively charged H^+^ ions in solution is high and they bind to the sawdust by electrostatic sorption, thereby competing with copper ions for binding sites. At low copper and zinc concentrations, the pH value of the solution increased, while the number of H^+^ ions decreased and the surface of the sorbents became negatively charged, resulting in increased Cu(II) and Zn(II) removal efficiency [78]. At higher pH values, the metal adsorption process stopped and hydroxide precipitation began [57].

For this reason, a favorable pH range for the sorption of heavy metal ions with sawdust exists [54,74]. Sciban and Krasnja [74] found that the optimum pH values for copper and zinc were 4.0 and 6.0, respectively, to avoid heavy metal precipitation.

The adsorption mechanism was confirmed by Rahman [79], who determined that the pH increased in the range of 2.0–8.0, and the removal efficiency of copper by maple sawdust increased from 28.66% to 83.25%.

### 3.4. Sorption Studies 

The related parameters calculated using the Freundlich and Langmuir equations at different concentrations of copper in solutions are summarized in Table 2 for every type of investigated sawdust. The adsorption of copper ions follows both Langmuir and Freundlich type isotherms.

**Figure 5:**
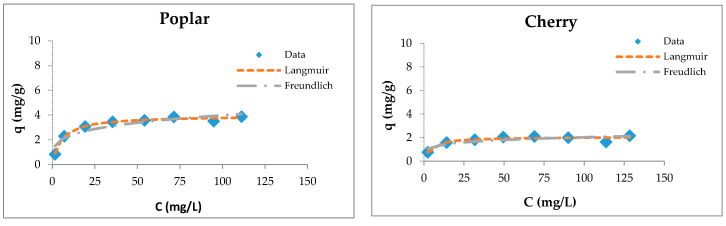

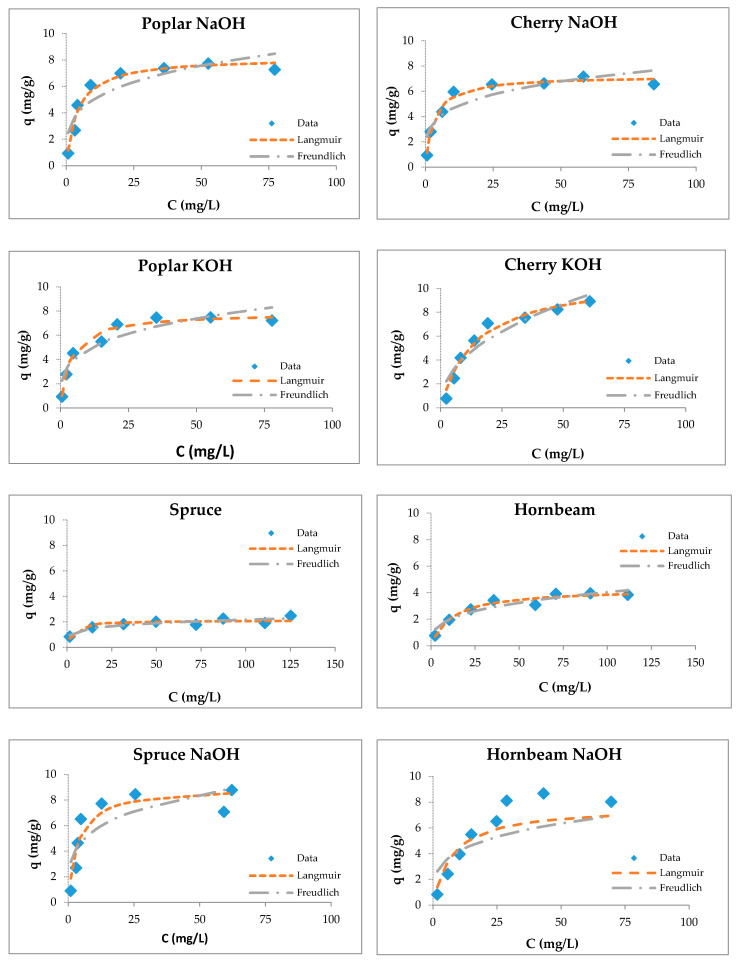

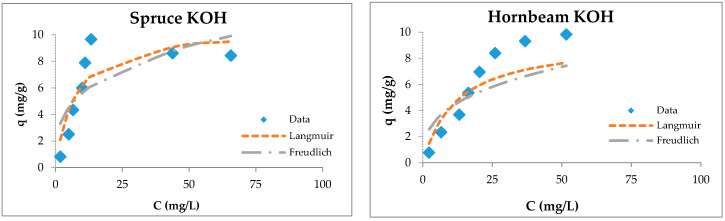
Langmuir and Freundlich isotherms of Cu(II) removal by raw and modified sawdusts.

The fitting of sorption experimental data for wooden sawdust is shown in Figure 5.

Although the four types of investigated sorbents are different in their anatomical structures and chemical compositions, they possess capacities for copper ion sorption. Langmuir isotherms are generally suitable for describing the chemisorption process when ionic or covalent chemical bonds are formed between the sawdust and the waters [74]. The sorption of copper by sawdust followed both types of adsorption isotherms; however, the Langmuir isotherm is more suitable for the description of its adsorption with poplar, cherry, and hornbeam sawdusts, as is evident from the values of the regression coefficient shown in Table 3. The values of the *R_L_* factor are between 0.235 and 0.590, indicating that the adsorption is favorable. This leads to the conclusion that the sorption process takes place as monolayer adsorption and that the surface of sawdust is homogenous in its adsorption affinity [55].

The sorption curves for zinc show the increase of the Zn(II) removal efficiency at low concentrations and then its decrease with the increase of the metal concentration. Due to this, the sawdust was saturated with a constant amount of zinc ions at higher concentrations, meaning that the number of active sites on the sorbent surface was limited [80].

**Figure 6:**
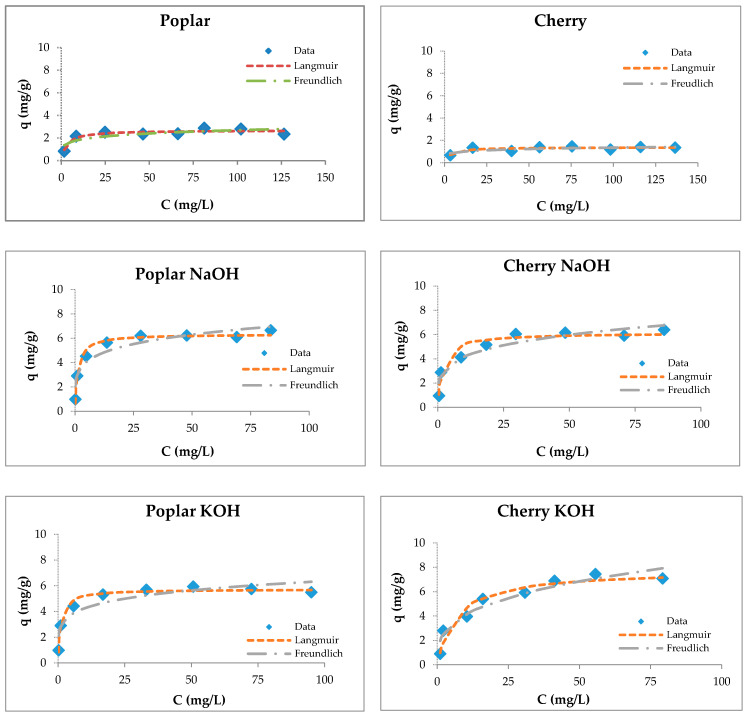

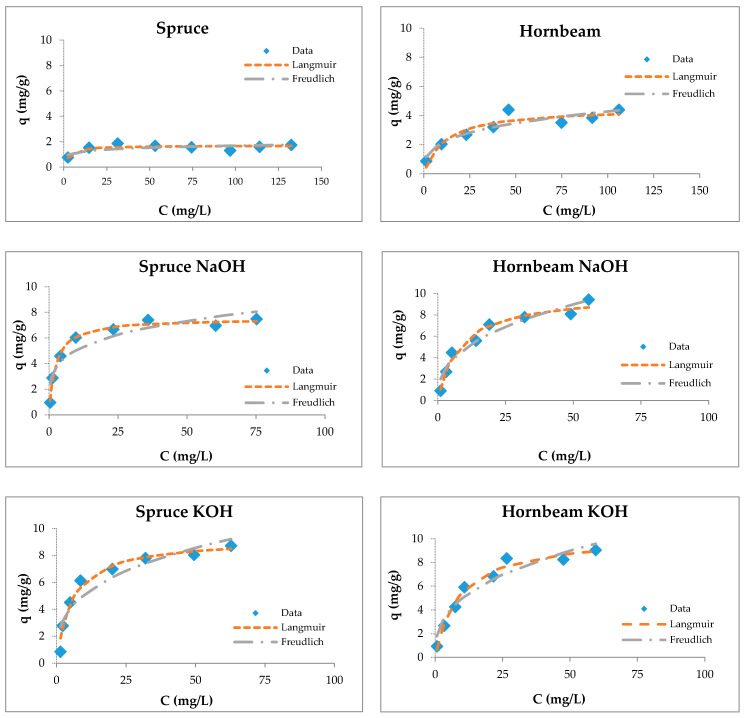
Langmuir and Freundlich isotherms of Zn(II) removal by raw and modified sawdusts.

The adsorption capacities of tested wooden sawdust for zinc ions were described by applying the corresponding equilibrium isotherms (Figure 6). The constants of sorption models are presented in Table 3. We can conclude (Table 3) that the correlation coefficients for the Freundlich model are smaller than those for the Langmuir model for all types of sawdust, except for natural spruce sawdust. The values of the *R_L_* factor lies between 0.093 and 0.537, which indicates that the adsorption is favorable. The modification with sodium hydroxide and potassium hydroxide did not change the surface of the sawdust, but improved the uptake of heavy metal ions due to the reaction of wooden sawdust with an alkaline solution, which caused the formation of new sorption sites on the sorbent surface [55,80,81,82,83].

The Langmuir isotherm fit better than the Feundlich model for sawdust sorbents, which was confirmed by another authors [34,37,57,74,84].

### 3.5. Maximum Sorption Capacities of Natural Sorbents

The maximum sorption capacity values obtained for tested wooden sawdusts and the literature data related to different low-cost sorbents used for removal of zinc and copper are shown in Table 4. The removal efficiencies of low-cost materials depend mainly on the wastewater parameters, for instance the pH, ionic strength, and temperature, as well as the sorbent properties (such as the specific surface area and surface chemistry). These factors can affect the stability of the heavy metals ions, together with the adsorptive characteristics of the materials used in the sorption process [3]. The sorption process depends on several parameters, such as the initial pH, initial concentration, adsorbent dosage, temperature, ionic strength, and particle size [54,55].

## 4. Conclusions

In this paper, we investigated the influence of hydroxide modification on the adsorption capacities of four types of sawdust. Wooden sawdust, which is a cheap and abundant material, was demonstrated to be an effective adsorbent for the removal of copper and zinc ions from model solutions. The adsorption efficiencies of poplar, cherry, spruce, and hornbeam sawdusts were almost the same for both metal ions, although they possess different chemical compositions and anatomical structures.

The treatment with sodium hydroxide and potassium hydroxide solutions increased the adsorption efficiency for both metals from 20% to 46.5%. The FTIR spectra confirmed an increase in the number of –OH functional groups for both NaOH- and KOH-modified sawdust. The isotherm data fit better with the Langmuir adsorption model compared to the Freundlich model for predicting the monolayer adsorption capacities for copper and zinc by natural and modified wooden sawdusts. The ion exchange or hydrogen binding mechanism well explained the zinc and copper adsorption by wooden sawdust. The highest removal efficiency was attained at 10 mg/L concentrations of copper and zinc in solution. Initially, the pH increased up to 5.3–6.0 for natural sawdust and 6.2–8.2 for modified sawdust and then slowly decreased, highlighting the adsorption mechanism. 

Wooden sawdust has the capacity to remove copper and zinc from synthetic solutions. Future research into wastewater and the applicability of wooden sawdust to the water treatment process is needed.

## Figures and Tables

**Table 1:** Description of heavy metal removal methods.

Techniques	Advantages	Disadvantages	Reference
Chemical precipitation	Simple procedure, inexpensive, useful for high concentrations of metals	Production of sludge, ineffective at low concentration of contaminants	[18,19]
Flotation	Selective metal ion recovery, low sludge generation, high separation efficiency	High cost	[20,21]
Ion exchange	No change in pH of wastewater, process reliability, stability, and chemical safety	High membrane cost, requirement of resin fouling, resin regeneration	[22,23]
Coagulation and flocculation	Settling and dewatering of sludge	High cost, large consumption of chemicals	[24,25]
Membrane filtration	Low production of solid waste, low consumption of chemicals, high efficiency	Low flow rate, high cost	[26,27]
Electrodialysis	Relatively low energy consumption, suitable for non-ionized from ionized components	Organic matter and colloids are not removed, elaborate controls are required, selection materials of construction membranes is important	[28,29]
Adsorption	Low formation of chemical and biological sludge, high efficiency, relatively low cost, regeneration of sorbents, and possibility of metal recovery	High cost of engineering sorbent	[7,30]

**Table 2:** Langmuir and Freundlich constants for copper sorption by wooden sawdust.

Wooden Sawdust	Langmuir Constants			Freundlich Constants		
	q_m_ (mg/g)	K_L_ (1/mg)	R^2^	K_F_ (1/g)	1/n	R^2^
Poplar	3.97 ± 0.26	0.19	0.99	1.35	0.23	0.87
Poplar NaOH	8.20 ± 1.43	0.24	0.96	2.75	0.26	0.82
Poplar KOH	7.86 ± 1.19	0.26	0.98	2.61	0.27	0.88
Cherry	2.08 ± 0.06	0.25	0.87	0.92	0.17	0.71
Cherry NaOH	7.38 ± 0.88	0.31	0.98	2.70	0.23	0.83
Cherry KOH	11.06 ± 1.93	0.07	0.97	1.54	0.44	0.90
Spruce	2.13 ± 0.05	0.32	0.79	0.88	0.20	0.84
Spruce NaOH	9.02 ± 1.73	0.28	0.87	3.21	0.25	0.68
Spruce KOH	10.52 ± 1.74	0.14	0.77	2.80	0.30	0.58
Hornbeam	4.32 ± 0.41	0.08	0.96	0.94	0.32	0.90
Hornbeam NaOH	7.71 ± 0.93	0.13	0.80	2.28	0.26	0.63
Hornbeam KOH	9.49 ± 1.04	0.08	0.78	1.95	0.34	0.65

**Table 3:** Langmuir constants for zinc sorption by wooden sawdust.

Wooden Sawdust	Langmuir Constants			Freundlich Constants		
	q_m_ (mg/g)	K_L_ (1/mg)	R^2^	K_F_ (1/g)	1/n	R^2^
Poplar	2.69 ± 0.08	0.35	0.89	1.27	0.16	0.69
Poplar NaOH	6.34 ± 0.47	0.82	0.978	3.00	0.19	0.90
Poplar KOH	5.73 ± 0.31	0.97	0.98	2.85	0.17	0.86
Cherry	1.38 ± 0.03	0.33	0.71	0.72	0.14	0.57
Cherry NaOH	6.15 ± 0.38	0.50	0.94	2.46	0.23	0.91
Cherry KOH	7.79 ± 0.96	0.14	0.95	1.96	0.32	0.93
Spruce	1.71 ± 0.04	0.35	0.71	0.86	0.15	0.78
Spruce NaOH	7.53 ± 0.98	0.42	0.99	2.97	0.23	0.87
Spruce KOH	9.26 ± 1.68	0.18	0.97	2.42	0.32	0.88
Hornbeam	4.54 ± 0.41	0.09	0.88	1.07	0.30	0.87
Hornbeam NaOH	10.02 ± 1.83	0.12	0.97	2.02	0.38	0.94
Hornbeam KOH	10.31 ± 1.71	0.11	0.98	2.13	0.37	0.93

**Table 4:** The maximum adsorbent capacity (*q_max_*) of different low-cost adsorbents.

Serial Number	Low-Cost Sorbent	q_max_ (mg/g)	Heavy Metals	Optimal pH	Reference
1	Tea Waste	8.9	Zn	4.2	[80]
2	Coffee residues	13.4 31.2	Zn Cu	-	[81]
3	Peanut shells	25.4	Cu	5.0	[82]
4	Banana peel	21.9 52.4	Zn Cu	4.0–6.0	[83]
5	Lemon peel	27.9 70.9	Zn Cu	4.0–6.0	[83]
6	Orange peel	27.1 63.3	Zn Cu	4.0–6.0	[83]
7	Peat (Danish)	34.1	Cu	4.0	[43]
8	Peat (Heilongjiang)	25.4	Cu	4.0	[43]
9	Poplar sawdust (raw)	0.74 0.86	Zn Cu	5.8 5.2	[55]
10	Beech sawdust	2.0 4.5	Zn Cu	4.8–5.3	[84]
11	Teakwood sawdust	4.9 11.0	Zn Cu	5.88 5.24	[85]
12	Meranti sawdust	32.1	Cu	6.0	[86]
13	Cherry sawdust (raw)	1.46 2.16	Zn Cu	5.1 4.1	Present study
14	Poplar sawdust (raw)	2.88 3.88	Zn Cu	5.1 4.4	Present study
15	Hornbeam sawdust (raw)	4.4 3.96	Zn Cu	5.4 4.1	Present study
16	Spruce sawdust (raw)	2.01 2.48	Zn Cu	5.6 4.2	Present study

## Supplementary Materials

The following are available online at https://www.mdpi.com/1996-1944/13/16/3575/s1, Figure S1: Infrared spectra of natural and treated spruce wooden sawdust, Figure S2: Infrared spectra of natural and treated hornbeam wooden sawdust.
