# Supplementary material for: Influence of Wooden Sawdust Treatments on Cu(II) and Zn(II) Removal from Water
Source: Materials (Basel). 2020 Aug 13;13(16):3575. doi: 10.3390/ma13163575 (PMC7476008; doi:10.3390/ma13163575)
Supplement: Supplementary file 1 [file materials-13-03575-s001.pdf]

materials

Supplementary Materials: Influence of Wooden Sawdust Treatments on Cu(II) and Zn(II) Removal from Water

Zdenka Kovacova [1], Stefan Demcak [1,*], Magdalena Balintova [1], Cocencepcion Pla [2] and Inga Zinicovscaia [3,4]

1 Faculty of Civil Engineering, Institute of Environmental Engineering, Technical University of Kosice, Vysokoskolska 4, 042 00 Kosice, Slovakia; zdenka.kovacova.2@tuke.sk (Z.K.); magdalena.balintova@tuke.sk (M.B.)
2 Department of Civil Engineering, University of Alicante, Carretera de s/n, 03690 Alicante, Spain; c.pla@ua.es
3 Joint Institute for Nuclear Research, Joliot-Curie Str., 6, 1419890 Dubna, Russia; zinikovskaia@mail.ru
4 Horia Hulubei National Institute for R&D in Physics and Nuclear Engineering, 30 Reactorului Str., MG-6, Bucharest, 077125 Magurele, Romania
* Correspondence: stefan.demcak@tuke.sk; Tel.: +421-155-602-4274

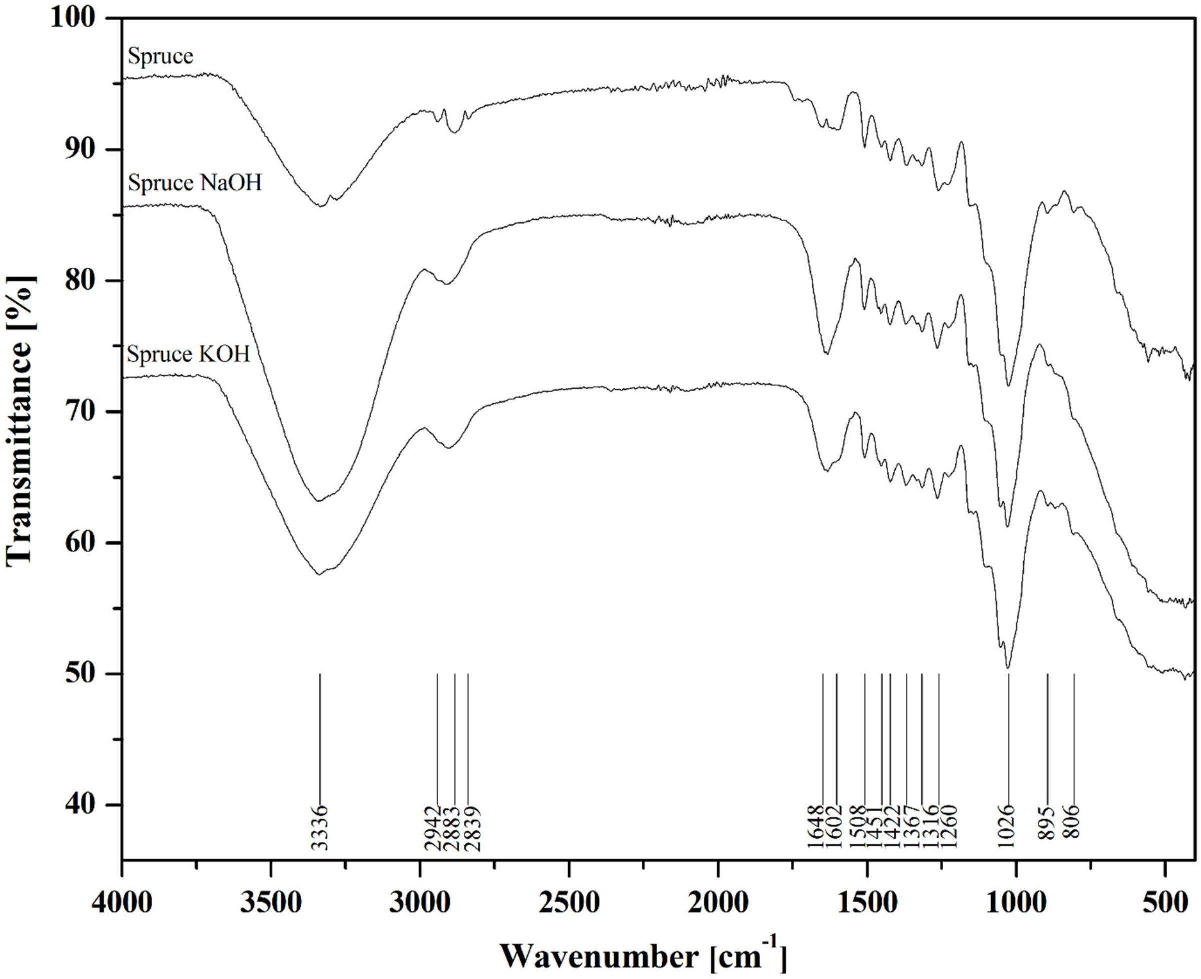

Figure S1. Infrared spectra of natural and treated spruce wooden sawdust.

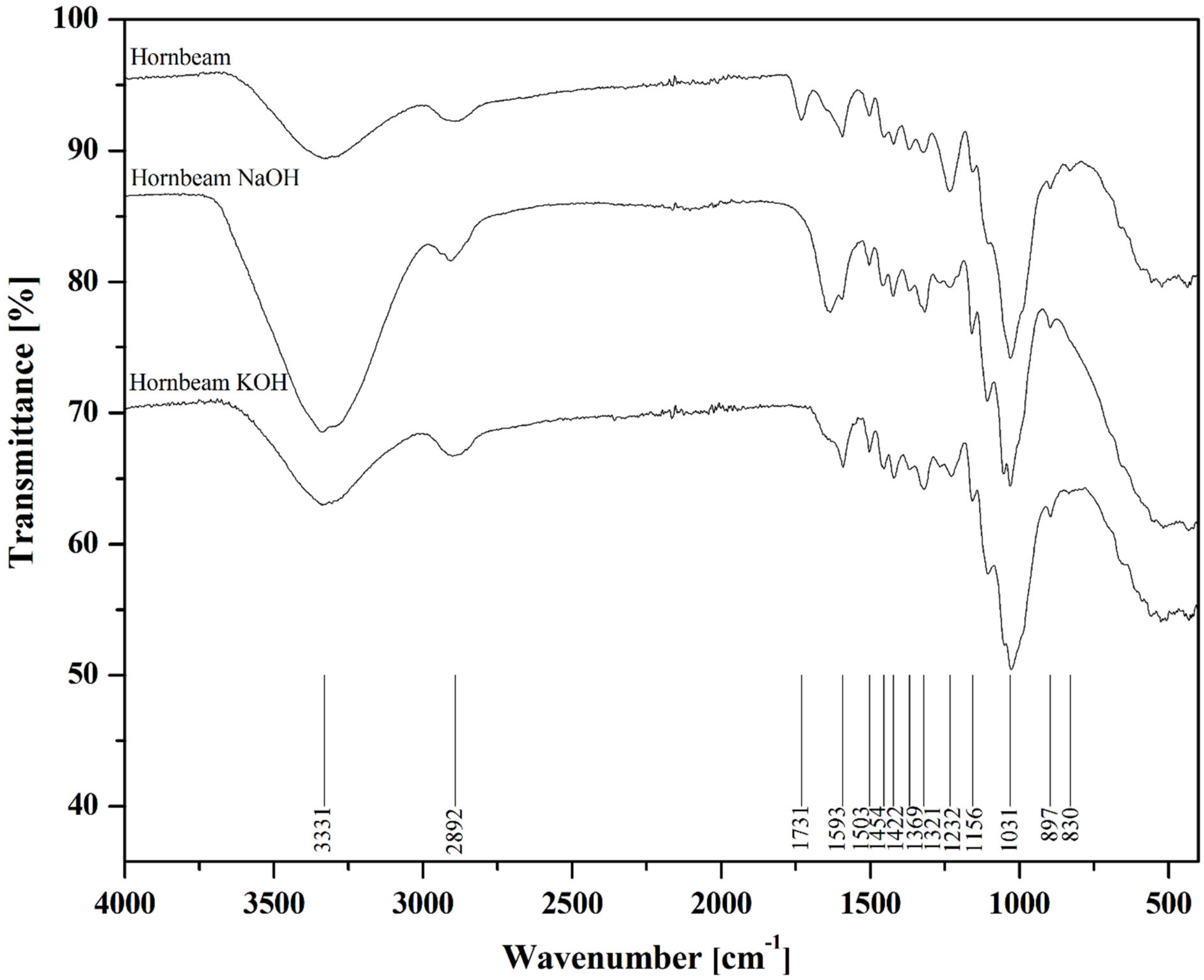

Figure S2. Infrared spectra of natural and treated hornbeam wooden sawdust.

© 2020 by the authors. Submitted for possible open access publication under the terms and conditions of the Creative Commons Attribution (CC BY) license (http://creativecommons.org/licenses/by/4.0/).
